# Outcomes of Patients with and without Malignancy Undergoing Percutaneous Pericardiocentesis for Pericardial Effusion

**DOI:** 10.3390/jcdd8110150

**Published:** 2021-11-06

**Authors:** Chun-Ting Shih, Wei-Chieh Lee, Hsiu-Yu Fang, Po-Jui Wu, Yen-Nan Fang, Shaur-Zheng Chong

**Affiliations:** 1 Division of Cardiology, Department of Internal Medicine, Kaohsiung Chang Gung Memorial Hospital and Chang Gung University College of Medicine, Kaohsiung 833401, Taiwan; u9801039@cmu.edu.tw (C.-T.S.); ast42aiu@hotmail.com (H.-Y.F.); sky1021@cgmh.org.tw (P.-J.W.); shauz@cgmh.org.tw (S.-Z.C.); 2 Institute of Clinical Medicine, College of Medicine, National Cheng Kung University, Tainan 701401, Taiwan

**Keywords:** pericardial effusion, malignancy, pericardiocentesis, prognosis, metastasis

## Abstract

(1) Background: This study aimed to evaluate the etiologies and clinical outcomes of patients with pericardial effusion (PE) treated with echo-guided percutaneous pericardiocentesis. (2) Methods: Between July 2010 and December 2020, a total of 502 patients underwent echo-guided percutaneous pericardiocentesis for PE at our hospital. The reasons for PE were malignancy (N = 277), and non-malignancy (N = 225). The comorbidities, complications, and all-cause mortality were compared between the malignancy and non-malignancy groups. (3) Results: In multivariable Cox regression analyses for 1-year mortality, malignancy related PE, nasopharyngeal and oropharyngeal cancer, and metastatic status were positive predictors. A higher incidence of in-hospital and 1-year mortality were observed in patients with malignancy-related PE than with non-malignancy-related PE. In patients with malignancy-related PE, the Kaplan-Meier curve of 1-year all-cause mortality significantly differed between patients with or without metastasis; however, PE with or without malignant cells did not influence the prognosis. (4) Conclusions: In the patients with large PE requiring percutaneous pericardiocentesis, malignancy-related PE, nasopharyngeal and oropharyngeal cancer, and metastatic status were positive predictors of 1-year mortality. In patients with malignancy, a higher incidence of all-cause mortality was noted in patients with metastasis but did not differ between the groups with and without malignant cells in PE.

## 1. Introduction

For patients with a large, symptomatic pericardial effusion (PE), percutaneous needle pericardiocentesis has been the most useful therapeutic procedure for the early management of cardiac tamponade, and it is also used as a diagnostic procedure in certain patients who required cytologic proof, even though the diagnostic yield is low [1,2,3,4]. In patients with cardiac tamponade, echocardiography is the main method for the diagnosis and evaluation of hemodynamic status. Echo-guided percutaneous needle pericardiocentesis can be performed after the selection of the most appropriate anatomical approach among the apical, subcostal, and parasternal approaches [5]. The major risks associated with percutaneous pericardiocentesis are chamber or coronary artery laceration requiring surgery, an injury to an intercostal vessel necessitating surgery, and pneumothorax which requires chest tube placement [6]. Moreover, the causes of PE have changed, and the prognosis of PE may differ over time due to an aging society [7,8].

PE commonly occurs in patients with malignancy and has been reported in up to 21% of patients, and lung cancer is the most common primary malignancy associated with PE, followed by breast cancer and lymphoma [9,10]. Malignancy is a common cause of PE and is a marker of poor prognosis. PE associated with malignancy may lead to cardiac tamponade, which is a life-threatening condition. Pericardial fluid drainage is typically performed in symptomatic patients and may play a role in its diagnosis and staging. Surgical drainage can be used for its management; however, it is associated with a high rate of morbidity [11]. In cancer patients with a poor prognosis, surgery may not be suitable as it involves high risk. Therefore, a less invasive strategy of echo-guided percutaneous needle pericardiocentesis presents the potential of the safe and effective management of these patients with cancer. However, the long-term outcomes of pericardiocentesis are less defined in the East Asian population.

For these reasons, this study aimed to evaluate the etiologies, clinical outcomes, and complications of patients with a large, symptomatic PE treated with echo-guided pericardiocentesis. 

## 2. Methods

### 2.1. Patient Population

**Figure 1:**
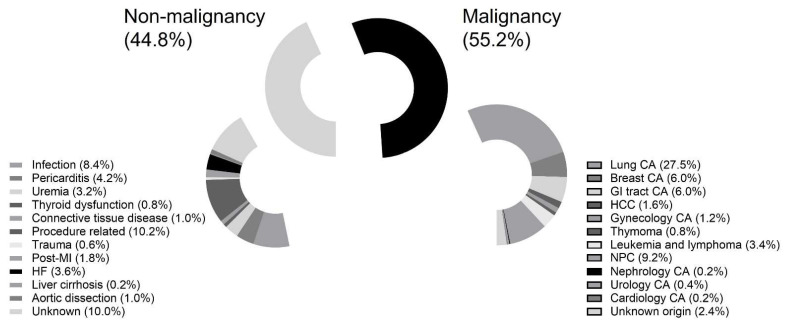
Patient eligibility. Abbreviations: CA: cancer; GI: gastrointestinal; HCC: hepatological cancer; NPC: nasopharyngeal cancer.

Between July 2010 and December 2020, a total of 502 patients underwent percutaneous pericardiocentesis for massive or symptomatic PE at our hospital. Patients were recruited if they underwent primary percutaneous pericardiocentesis and were excluded if they underwent pericardial window surgery. The reasons for PE include malignancy (N = 277; 55.2%), infection (N = 42; 8.4%), pericarditis (N = 21; 4.2%), uremia (N = 16; 3.2%), thyroid dysfunction (N = 4; 0.8%), connective tissue disease (N = 5; 1.0%), procedure-related (N = 51; 10.2%), trauma (N = 3; 0.6%), unknown (N = 50; 10.0%), post-myocardial infarction (N = 9; 1.8%), heart failure (N = 18; 3.6%), liver cirrhosis (N = 1; 0.2%), and aortic dissection (N = 5; 1.0%) (Figure 1). 

The comorbidities, laboratory data, prior cancer therapy (including chemotherapy, or radiation therapy), clinical symptoms (dyspnea, tachycardia, and shock), echocardiographic findings, color and laboratory data of PE, complications, and 1-year all-cause mortality were compared between the malignancy and non-malignancy groups. The effusion pathology and microbiological results obtained at the time of pericardiocentesis were reviewed. The 1-year all-cause mortality was compared between subgroups of the malignancy group, including those with and without metastasis, with or without malignant cells in PE, with or without recurrent malignancy status, and between different cancers. 

### 2.2. Percutaneous Pericardiocentesis Procedures

Percutaneous pericardiocentesis was performed under echocardiographic guidance at the shortest distance to the pericardial cavity from the apical, subcostal, and parasternal sites. Both the needle and catheter positions were confirmed using a saline contrast injection via echocardiography. After accessing the pericardial space, the needle was exchanged over a wire to a dilator, followed by a multiple side-hole pigtail catheter. The catheter was sutured and fixed to the chest wall. A PE sample (50 mL) of aspirated fluid was sent for pathology, chemistry, and microbiological testing. Post-procedure chest radiographs were performed regularly to assess the catheter position and any immediate complications. The catheter was removed at an earlier stage if fluid drainage fell below 50 mL per day and if no residual effusion was observed during follow-up echocardiography. 

### 2.3. Definition

Cardiac tamponade was diagnosed by observing the compression of the right ventricle in diastole using echocardiography, in the presence of tachycardia (heart rate > 100 beats per min) or pulsus paradoxus (>10 mmHg decreases in the systolic blood pressure on inspiration) [12]. A large PE was diagnosed after the detection of the distance of the echo-free space of >2 cm on echocardiography [13]. All-cause mortality was defined as death resultant of any cause. 

### 2.4. Study Endpoint

The study endpoint was all-cause mortality at a 1-year follow-up. 

### 2.5. Statistical Analysis

Data are presented as mean ± standard deviation, or median ± quartile deviation if they are non-normally distributed parameters or numbers (percentages). The characteristics of the study groups were compared using the *t*-test for continuous variables and the chi-square test for categorical variables. Univariable and multivariable Cox regression analyses for one-year mortality were both performed to identify significant determinants. The multivariable Cox regression analysis included a hazard ratio (HR) <0.100 for one-year mortality in the univariable Cox regression analyses. Kaplan–Meier curves were created to illustrate the 1-year all-cause mortality data for each group. A statistical analysis was performed using statistical software (SPSS for Windows, version 22, IBM. Corp., Armonk, NY, USA), and a two-sided *p*-value of < 0.05 was defined as statistically significant. 

## 3. Results

### 3.1. Baseline Characteristics of the Study Patients

The baseline characteristics of the study population are presented in Table 1. All patients underwent a percutaneous pericardiocentesis of a large PE with or without cardiac tamponade. The mean age was 63 ± 13.4 years, and most patients were males (61.6%). The participants in the malignancy group were younger than those in the non-malignancy group (59 ± 12.3 years vs. 67 ± 15.3 years; *p* < 0.001). In the non-malignancy group, a higher prevalence of diabetes mellitus (32.9% vs. 18.8%; *p* < 0.001), hypertension (32.9% vs. 18.8%; *p* < 0.001), coronary artery disease (32.9% vs. 5.1%; *p* < 0.001), and end-stage renal disease (16.4% vs. 4.0%; *p* < 0.001) were observed than in the malignancy group. A prior history of pericardiocentesis was higher in the malignancy group than in the non-malignancy group (9.7% vs. 4.0%; *p* = 0.014).

A higher white blood cell count (WBC) (9.6 ± 4.0 × 10^3^/μL vs. 10.8 ± 6.8 × 10^3^/μL; *p* = 0.010) and higher levels of lactate dehydrogenase (242 ± 246 mg/dL vs. 284 ± 288 mg/dL; *p* = 0.016) were observed in the malignancy group. A higher prevalence of asymptomatic participants in the non-malignancy group was documented (18.2% vs. 10.1%; *p* = 0.009). Furthermore, a higher prevalence of combined pleural effusion (78.7% vs. 60.4%; *p* < 0.001), cardiac tamponade (88.8% vs. 75.6%; *p* < 0.001), and low voltage on electrocardiography (42.0% vs. 25.4%; *p* < 0.001) were observed in the malignancy group. The need for subsequent pericardial peritoneal window surgery did not differ between the two groups. The incidence of complications related to pericardiocentesis was similar between the two groups. The complications included right ventricular laceration (1), left ventricular laceration (1), pneumothorax (1), and mixed pleural space insertion (2). 

A higher incidence of in-hospital mortality (46.9% vs. 14.7%; *p* < 0.001) and all-cause mortality (56.3% vs. 18.7%; *p* < 0.001) were observed in the malignancy group. A shorter median of the follow-up duration (106 ± 107 days vs. 630 ± 636 days; *p* < 0.001) was observed in the malignancy group. 

### 3.2. The Type of Malignancy and Associated Mortality Rate 

Most of the original sites in the malignancy group were lung cancer (49.8%), nasopharyngeal and oropharyngeal cancer (16.6%), breast cancer (10.8%), and gastrointestinal tract cancer (10.8%) (Table 2). Of the total participants in the malignancy group, 6.5% had double cancer. A total of 76.2% of participants had metastasis, and 16.6% had recurrent status. Over half of the participants (59.2%) received prior chemotherapy and/or radiotherapy. A total of 35.4% of the participants’ PE samples contained malignant cells. 

**Figure 2:**
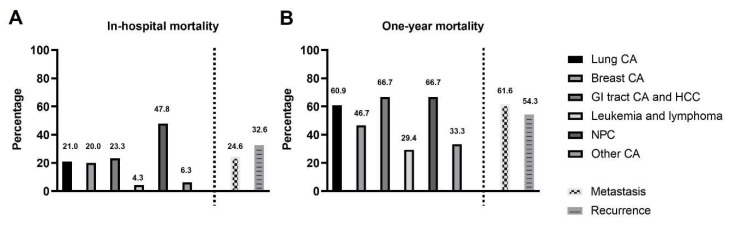
(**A**): The incidence of in-hospital mortality in different cancers: The patients with nasopharyngeal and oropharyngeal cancer had the highest in-hospital mortality rate. (**B**). The incidence of 1-year all-cause mortality in different cancers: The patients with gastrointestinal tract cancer and nasopharyngeal and oropharyngeal cancer had the highest 1-year mortality rate. Abbreviations: CA: cancer; GI: gastrointestinal; HCC: hepatological cancer; NPC: nasopharyngeal cancer.

In our study, patients with nasopharyngeal and oropharyngeal cancer had the highest in-hospital mortality rate (Figure 2A). Patients with gastrointestinal tract cancer and nasopharyngeal and oropharyngeal cancer had the highest incidence of 1-year mortality (Figure 2B). 

### 3.3. Univariable and Multivariable Cox Regression Analyses of Predictors of One-Year Mortality 

Hypertension, liver cirrhosis, coronary artery disease, end-stage renal disease, procedure related malignancy, lung cancer, gastrointestinal tract cancer and hepatological cancer, nasopharyngeal cancer, metastatic and recurrent status, malignant cells in PE, symptoms including dyspnea, tachycardia, and pleural effusion were included for the multivariable Cox regression analyses of one-year mortality incidence (Table 3). Malignancy (HR: 2.084; 95% confidence interval (CI): 1.246–3.488; *p* = 0.005), nasopharyngeal cancer (HR: 1.801; 95% CI: 1.194–2.717; *p* = 0.005), and the metastatic status (HR: 2.088; 95% CI: 1.352−3.223; *p* = 0.001) were independent predictors of one-year mortality (Table 3).

### 3.4. Kaplan–Meier Curves Showing 1-Year All-Cause Mortality Data of the Two Groups and Subgroups of the Malignancy Population

**Figure 3:**
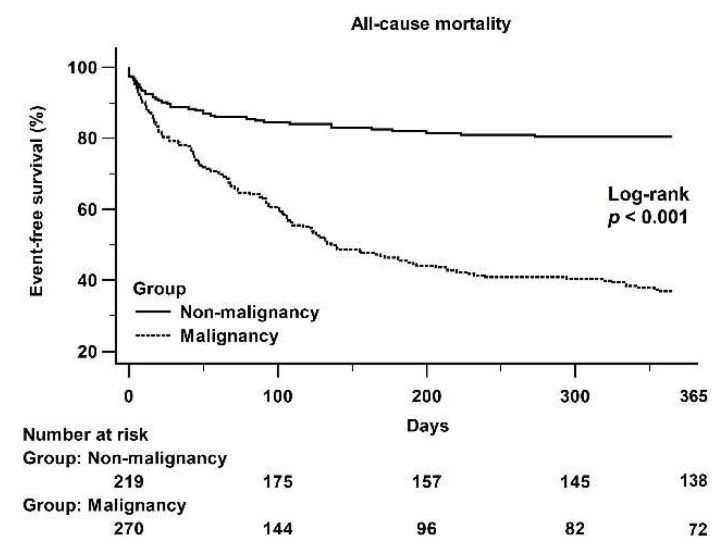
A Kaplan–Meier curve of the 1-year all-cause mortality between the non-malignancy and malignancy groups: There was a significant difference between the non-malignancy and malignancy groups (log-rank *p* < 0.001).

Six patients in the non-malignancy group and seven patients in the malignancy group died within a day of pericardiocentesis. Figure 3 shows a Kaplan–Meier curve that illustrates the difference in 1-year all-cause mortality between the non-malignancy and malignancy groups (log-rank *p* < 0.001). 

**Figure 4:**
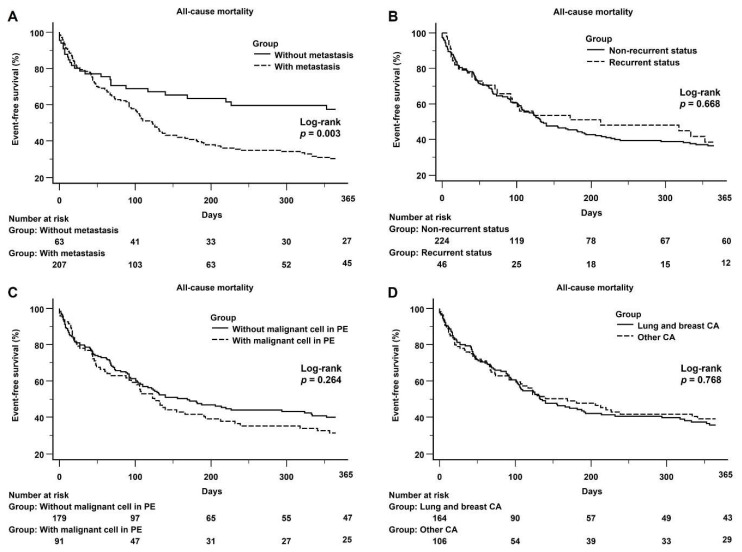
(**A**): Kaplan–Meier curve of the 1-year all-cause mortality between the subgroups with and without metastasis: There was a significant difference between the subgroups with and without metastasis (log-rank *p* = 0.003). (**B**): Kaplan–Meier curve of the 1-year all-cause mortality between the subgroups with recurrent and non-recurrent status: There was no significant difference between the subgroups with and without metastasis (log-rank *p* = 0.668). (**C**): Kaplan–Meier curve of the 1-year all-cause mortality between the subgroups with and without malignancy in PE: There was no significant difference between the subgroups with and without malignancy in PE (log-rank *p* = 0.264). (**D**): Kaplan–Meier curve of the 1-year all-cause mortality between lung and breast cancer and other cancers, no significant difference between lung and breast cancer and other cancers was observed (log-rank *p* = 0.768).

In the malignancy group, there was a significant difference in 1-year all-cause mortality between the subgroups, both with and without metastasis (log-rank *p* = 0.003) (Figure 4A). A significant difference in the 1-year all-cause mortality between the recurrent and non-recurrent (Figure 4B) was not observed, and with or without the presence of malignant cells in PE (Figure 4C). Figure 4D shows that there was not a significant difference in the 1-year all-cause mortality between lung and breast cancer and other cancers. 

## 4. Discussion

In the present study, a low incidence of complications (1.0%) was noted for the entire study population; however, two patients required surgery for cardiac ventricular laceration. The incidence of all-cause mortality was significantly higher in the malignancy group than in the non-malignancy group. Most of the malignancies were associated with lung cancer (49.8%), nasopharyngeal and oropharyngeal cancer (16.6%), breast cancer (10.8%), and gastrointestinal tract cancer (10.8%). Of all the participants, 76.2% were of a metastatic status, and 59.2% had received chemotherapy and/or radiotherapy. Participants with metastatic malignancy also presented poorer outcomes than those without metastatic malignancy. The prognosis did not differ between direct invasion (lung and breast cancer) and non-direct invasion (other types of malignancy). In our study, cytology, with or without malignant cells in the PE sample, did not influence the prognosis. Patients with nasopharyngeal and oropharyngeal cancer had the highest in-hospital mortality rate. Patients with gastrointestinal tract cancer and nasopharyngeal and oropharyngeal cancer had the highest 1-year mortality rate. Malignancy related PE, nasopharyngeal and oropharyngeal cancer, and metastatic status were positive predictors of one-year mortality. 

### 4.1. Poor Prognosis in Malignant PE

Over time, the causes of large PE, with or without cardiac tamponade, changed from iatrogenic problems to malignancy, and patients with malignant PE had poorer outcomes [4,8]. In our study, the median survival duration was significantly shorter in the malignancy population than in the non-malignancy population (106 ± 107 days vs. 630 ± 636 days; *p* < 0.001). Currently, the prolonged survival rate of patients with cancer may lead to additional problems, such as malignant PE. Echo-guided percutaneous pericardiocentesis with drainage is a safe and effective treatment for patients with a large and symptomatic PE and can improve symptoms and quality of life but does not prolong life [14]. Therefore, a conservative strategy or hospice care may be considered for patients with malignant PE who have a shorter survival duration, especially for patients with nasopharyngeal and oropharyngeal cancer, and metastatic status.

### 4.2. The Predictors of Mortality in the Cancer Population with Malignant PE

In our study, patients with a metastatic status had a poor prognosis; however, the occurrence of malignant cells in PE did not differ between the groups. Malignant cells in PE presented a significant in univariate analysis but did not constitute a positive predictor in the multivariate Cox regression analyses. Previous reports have stated that the presence of pericardial malignant cytology does not appear to significantly affect outcomes [8]. However, another study reported that pericardial malignant cytology can be used to predict poor clinical outcomes in patients with malignant PE [15]. Lekhakul reported that patients with either lymphoma or chronic leukemia presented better survival than those with carcinoma or sarcoma [14]. El Haddad found that malignant PE significantly shortens the survival outcome of patients with lung cancer, but not of patients with breast cancer [10]. Therefore, about malignant cytology in PE remains controversial, regarding whether it results in poor outcomes and which cancer has a worse prognosis in cancer patients with malignant PE. In our study, the prognosis did not differ between different types of cancer; however, cancer patients with a metastatic status had a poorer prognosis.

### 4.3. Study Limitations

One limitation of this study is its retrospective nature, and that it included data from only one medical center. As a retrospective chart review, decisions regarding the method, entry site and placement of the catheter for extended drainage were all dependent on the operator and the patient’s general status (possible selection bias). Our patient population’s initial performance status could not be obtained from the data collected. The benefits of symptom relief or of improved short-term quality of life after pericardiocentesis cannot be measured, especially in patients with terminal stage disease. However, we have provided important information with regard to a comparison between patients with malignant and non-malignant PE, and we reported the poor prognosis of cancer patients with malignant PE in the East-Asian population, especially in patients with nasopharyngeal and oropharyngeal cancer.

## 5. Conclusions

In the patients with a large PE requiring percutaneous pericardiocentesis, malignancy related PE, nasopharyngeal and oropharyngeal cancer, and metastatic status were positive predictors of one-year mortality. A higher incidence of all-cause mortality was noted in patients with metastasis, although no differences in mortality were observed between the groups with and without malignant cells in PE.

## Figures and Tables

**Table 1:** Demographics and clinical characteristics.

Variables	All Patients (N = 502)	Non-Malignancy (N = 225)	Malignancy (N = 277)	*p* Value
*Demographic*				
Age (years)	63 ± 13.4	67 ± 15.3	59 ± 12.3	<0.001
Male gender (%)	309 (61.6)	150 (66.7)	159 (57.4)	0.034
*Medical history*				
Diabetes mellitus (%)	126 (25.1)	74 (32.9)	52 (18.8)	<0.001
Hypertension (%)	213 (42.4)	140 (62.2)	73 (26.4)	<0.001
Connective disease (%)	10 (2.0)	7 (3.1)	3 (1.1)	0.121
Liver cirrhosis (%)	28 (5.6)	12 (5.3)	16 (5.8)	0.848
CAD (%)	88 (17.5)	74 (32.9)	14 (5.1)	<0.001
Heart failure (%)	15 (3.0)	11 (5.2)	4 (1.7)	0.062
ESRD (%)	48 (9.6)	37 (16.4)	11 (4.0)	<0.001
Thyroid dysfunction (%)	72 (26.5)	33 (14.7)	39 (14.1)	0.898
Prior history of pericardiocentesis (%)	36 (7.2)	9 (4.0)	27 (9.7)	0.014
*Lab data*				
WBC (1000/μL)	10.3 ± 5.7	9.6 ± 4.0	10.8 ± 6.8	0.010
Hgb (g/dL)	11.1 ± 2.1	11.0 ± 2.2	11.2 ± 2.1	0.153
PLT (1000/μL)	227 ± 111	220 ± 108	233 ± 112	0.221
Creatinine (excluded ESRD) (mg/dL)	1.36 ± 1.24	1.64 ± 0.66	1.16 ± 0.76	<0.001
LDH (mg/dL)	267 ± 267	242 ± 246	284 ± 288	0.016
Glucose (mg/dL)	147.3 ± 57.7	141.7 ± 57.5	151.7 ± 57.6	0.158
*Clinical symptoms*				
asystematic	69 (13.7)	41 (18.2)	28 (10.1)	0.009
dyspnea	345 (68.7)	136 (60.4)	209 (75.5)	<0.001
tachycardia	408 (81.3)	163 (72.4)	245 (88.4)	<0.001
shock	191 (38.0)	92 (40.9)	99 (35.7)	0.267
*Pleural effusion*				
Combined	354 (70.5)	136 (60.4)	218 (78.7)	<0.001
Bilateral side	177 (35.3)	63 (28.0)	114 (41.2)	0.001
Only left side	133 (26.5)	64 (87.7)	69 (66.3)	
Only right side	44 (8.8)	9 (12.3)	35 (33.7)	
*Ascites*	69 (13.7)	37 (16.4)	32 (11.6)	0.120
*Echo*				
Cardiac tamponade (%)	416 (82.9)	170 (75.6)	246 (88.8)	<0.001
*Low voltage on ECG*	173 (34.5)	57 (25.4)	116 (42.0)	<0.001
*Pericardial effusion*				
The color				<0.001
Fresh red	143 (28.5)	66 (29.3)	77 (27.8)	
Dark red	178 (35.5)	66 (29.3)	112 (40.4)	
Yellow	139 (27.7)	60 (26.7)	79 (28.5)	
Turbid	42 (8.4)	33 (14.7)	9 (3.2)	
Lab data				
WBC (/μL)	1470 ± 1476	1435 ± 1322	1530 ± 1745	0.442
LDH (mg/dL)	566 ± 572	462 ± 474	666 ± 674	0.758
Glucose (mg/dL)	108 ± 108	115 ± 117	96 ± 98	0.082
The mean of drainage amount (mL)	376.1 ± 253.1	356.9 ± 274.8	389.0 ± 237.0	0.194
*Surgery of pericardial periotoneal window (%)*	30 (6.0)	9 (4.1)	21 (7.7)	0.129
*Complications of pericardiocentesis (%)*	5 (1.0)	4 (1.8)	1 (0.4)	0.179
*Clinical outcomes*				
In-hospital mortality (%)	163 (32.5)	17 (7.6)	66 (23.8)	<0.001
One-year mortality (%)	198 (39.4)	42 (18.7)	156 (56.3)	<0.001
*Median of F/U duration (days)*	206 ± 222	630 ± 636	106 ± 107	<0.001

Data are expressed as mean ± standard deviation or median ± quartile deviation or as a number (percentage). Abbreviations: CAD: coronary artery disease; ESRD: end-stage renal disease; WBC: white blood cell; Hgb: hemoglobin; PLT: platelet; LDH; lactic acid dehydrogenase; F/U: follow-up.

**Table 2:** Type of malignancy.

Variables	All Patients (N = 277)
*Original site*	
Lung (%)	138 (49.8)
Breast (%)	30 (10.8)
GI tract (%)	30 (10.8)
Hepatology (%)	8 (2.9)
Gynecology (%)	6 (2.2)
Thymoma (%)	4 (1.4)
Leukemia and lymphoma (%)	17 (6.1)
Nasopharyngeal and oropharyngeal (%)	46 (16.6)
Nephrology (%)	1 (0.4)
Urology (%)	2 (0.7)
Cardiology (%)	1 (0.4)
Unknown (%)	12 (4.3)
*Double cancer (%)*	18 (6.5)
*Metastasis*	211 (76.2)
Lung/pleural	181 (65.3)
Pericardial	110 (39.7)
Extrathoracic	125 (45.1)
*Recurrence*	46 (16.6)
*Prior history of chemotherapy and/or radiotherapy*	164 (59.2)
*Malignancy in pericardial effusion*	98 (35.4)

Data are expressed as a number (percentage). Abbreviations: GI: gastrointestinal.

**Table 3:** Univariable and multivariable Cox regression analyses of predictors of one-year mortality.

	Univariate Analysis			Multivariate Analysis		
Variables	HR	95% CI	*p* value	HR	95% CI	*p* value
Age	0.994	0.984−1.003	0.188			
Male gender	0.884	0.666−1.173	0.393			
Diabetes mellitus	0.781	0.559−1.092	0.149			
Hypertension	0.497	0.368−0.673	<0.001			
Connective disease	0.429	0.107−1.729	0.234			
Liver cirrhosis	1.797	1.107−2.917	0.018			
CAD	0.405	0.252−0.651	<0.001			
Heart failure	1.140	0.505−2.577	0.752			
ESRD	0.578	0.330−1.015	0.057			
Thyroid dysfunction	0.976	0.651−1.465	0.908			
Procedure related	0.445	0.235−0.841	0.013			
Malignancy	4.055	2.879−5.712	<0.001	2.084	1.246–3.488	0.005
Lung CA	2.348	1.769−3.117	<0.001			
Breast CA	1.250	0.726−2.152	0.421			
GI tract CA and HCC	1.818	1.166−2.832	0.008			
Leukemia and lymphoma	0.917	0.407−2.066	0.834			
NPC	2.490	1.685−3.680	<0.001	1.801	1.194–2.717	0.005
Metastasis	3.565	2.651−4.794	<0.001	2.088	1.352–3.223	0.001
Recurrence	1.593	1.047−2.423	0.030			
Prior history of pericardiocentesis	1.388	0.865−2.227	0.174			
WBC of PE (1000/μL)	1.000	0.997−1.002	0.572			
LDH of PE (mg/dL)	1.000	0.998−1.003	0.865			
Glucose of PE (mg/dL)	1.001	1.000−1.001	0.206			
Malignant cells in PE	2.184	1.606−2.969	<0.001			
Dyspnea	1.484	1.076−2.045	0.016			
Tachycardia	2.029	1.313−3.135	0.001			
Shock	0.952	0.713−1.271	0.739			
Pleural effusion	2.223	1.548−3.192	<0.001			
Ascites	0.984	0.660−1.468	0.937			
Cardiac tamponade	1.397	0.931−2.098	0.106			
Drainage amount	1.000	0.999−1.000	0.287			
Complications of pericardiocentesis	0.049	0−19.601	0.324			

Abbreviation: HR: hazard ratio; CI: confidence interval; CAD: coronary artery disease; ESRD: end-stage renal disease; CA: cancer; HCC: hepatology cancer; NPC: nasopharyngeal cancer; WBC: white blood cell; LDH: lactate dehydrogenase; PE: pericardial effusion.

## Data Availability

The data of this study are available from the corresponding author upon request.
